# Supplementary material for: Instantly adhesive and ultra-elastic patches for dynamic organ and wound repair
Source: Nat Commun. 2024 Jun 3;15:4720. doi: 10.1038/s41467-024-48980-0 (PMC11148085; doi:10.1038/s41467-024-48980-0)
Supplement: Supplementary file 1 — Supplementary Information [file 41467_2024_48980_MOESM1_ESM.pdf]

Supplementary Information

Instantly adhesive and ultra-elastic patches for dynamic organ and wound repair

Parth Chansoria[1,†], Ameya Chaudhari[1,†], Emma L. Etter[1], Emily E. Bonacquisti[1], Mairead K. Heavey[1], Jiayan Le[1], Murali Kannan Maruthamuthu[1], Caden C. Kussatz[1], John Blackwell[2], Natalie E. Jasiewicz[1], Rani S. Sellers[3], Robert Maile[4,5,], Shannon M. Wallet[6], Thomas M. Egan[2,7], Juliane Nguyen[1,7*]

[1]Division of Pharmacoengineering and Molecular Pharmaceutics, Eshelman School of Pharmacy, University of North Carolina at Chapel Hill, Chapel Hill, NC 27599, USA

[2]Division of Cardiothoracic Surgery, Department of Surgery, School of Medicine, University of North Carolina at Chapel Hill, Chapel Hill, NC 27599, USA

[3]Pathology and Laboratory Medicine, Department of Medicine, University of North Carolina, Chapel Hill, NC 27599, USA

[4]Department of Surgery, University of North Carolina at Chapel Hill, Chapel Hill, NC 27599, USA

[5]Department of Microbiology and Immunology, School of Medicine, University of North Carolina at Chapel Hill, Chapel Hill, NC 27599, USA

[6]Division of Oral and Craniofacial Health Sciences, University of North Carolina at Chapel Hill, Chapel Hill, NC 27599, USA

[7]Joint Department of Biomedical Engineering, University of North Carolina at Chapel Hill, Chapel Hill, NC 27599, and North Carolina State University, Raleigh, NC, 27695, USA

[†]- These authors contributed equally

*Correspondence: julianen@email.unc.edu (Juliane Nguyen)

This PDF includes:

Supplementary Figures 1 and 6

Supplementary Tables 1 to 3

Other Supplementary Materials for this manuscript include the following:

Supplementary Movies 1 to 10

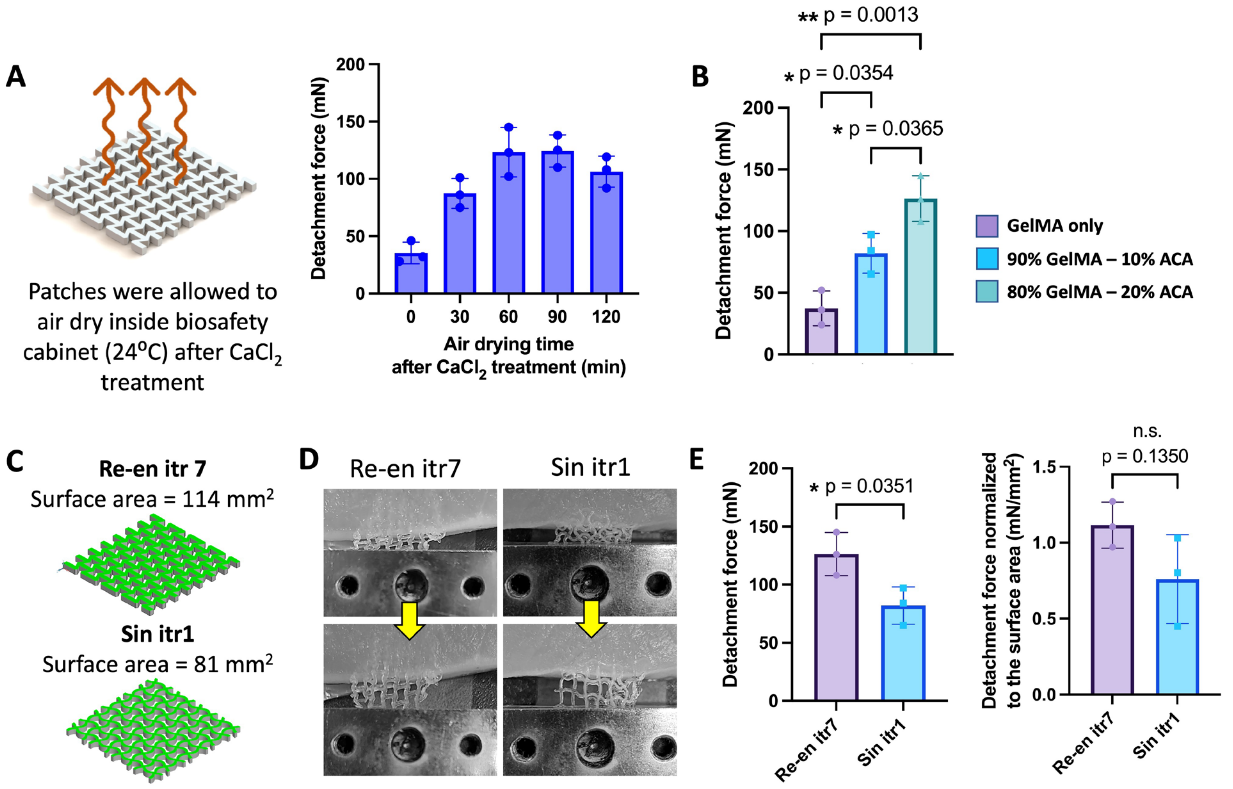

Supplementary Figure 1. A. Air drying of AuxES patches after $CaCl_2$ treatment improves the adhesiveness. **B.** The detachment force increased upon increasing the ACA content up to 20%, which can be attributed to increased ionic interactions via Ca^{2+} bridging and hydrogen bonding between the patch and the tissue. Statistical significance was analyzed by one-way ANOVA followed by Tukey's multiple comparison tests,$*p < 0.05$, and $**p < 0.01$. **C.** Comparison of the bioadhesiveness of the different patch architectures, where Re-en itr7 and Sin itr1 architectures (15×15 mm^2) were selected. **D.** Adhesiveness of the patches was compared in a tensile testing setup. **E.** Re-en itr7 patches with higher surface area of attachment demonstrated a higher attachment force compared to the Sin itr1 patches, but the force normalized to the surface area was not significantly different. Differences in the means of the normalized detachment force could be attributed to the higher elasticity of the Re-ent itr7 patches compared to the Sin itr1 patches. Statistical significance was analyzed by a two-tailed t-test, $*p < 0.05$. Data in A, B, and E are from n=3 biologically independent samples and are presented as mean ± s.d.

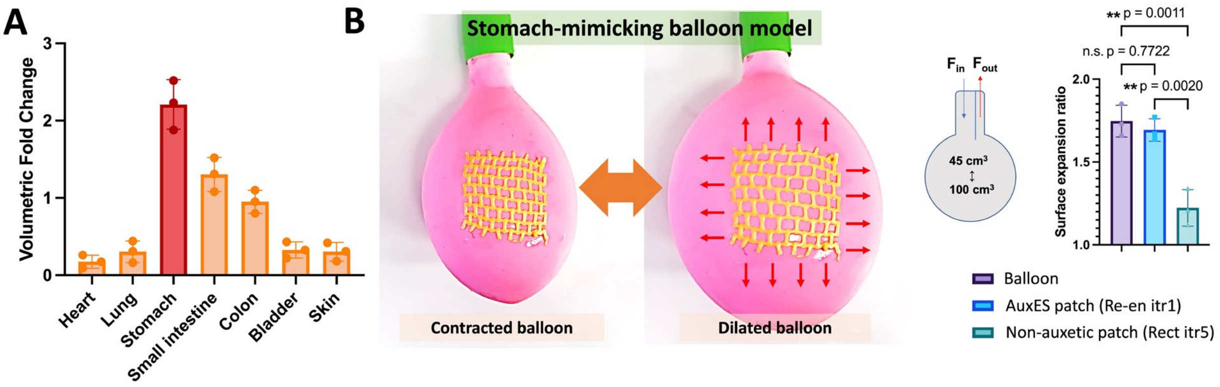

Supplementary Figure 2. A. Volumetric fold change of different dynamic organs. The stomach has the highest volumetric fold change compared with other dynamic organs.[1] **B.** The AuxES patch (Re-en itr1 architecture) tested on a dilating balloon model demonstrated adherence to the balloon mechanics and a similar surface expansion to that of the balloon (see **Supplementary Movie 4**), while the non-auxetic patch (Rect itr5) did not conform to the

deformation of the balloon. Statistical significance was analyzed by one-way ANOVA followed by Tukey's multiple comparison tests,*p < 0.05, and **p < 0.01. Data in B are from n=3 biologically independent samples and are presented as mean ± s.d.

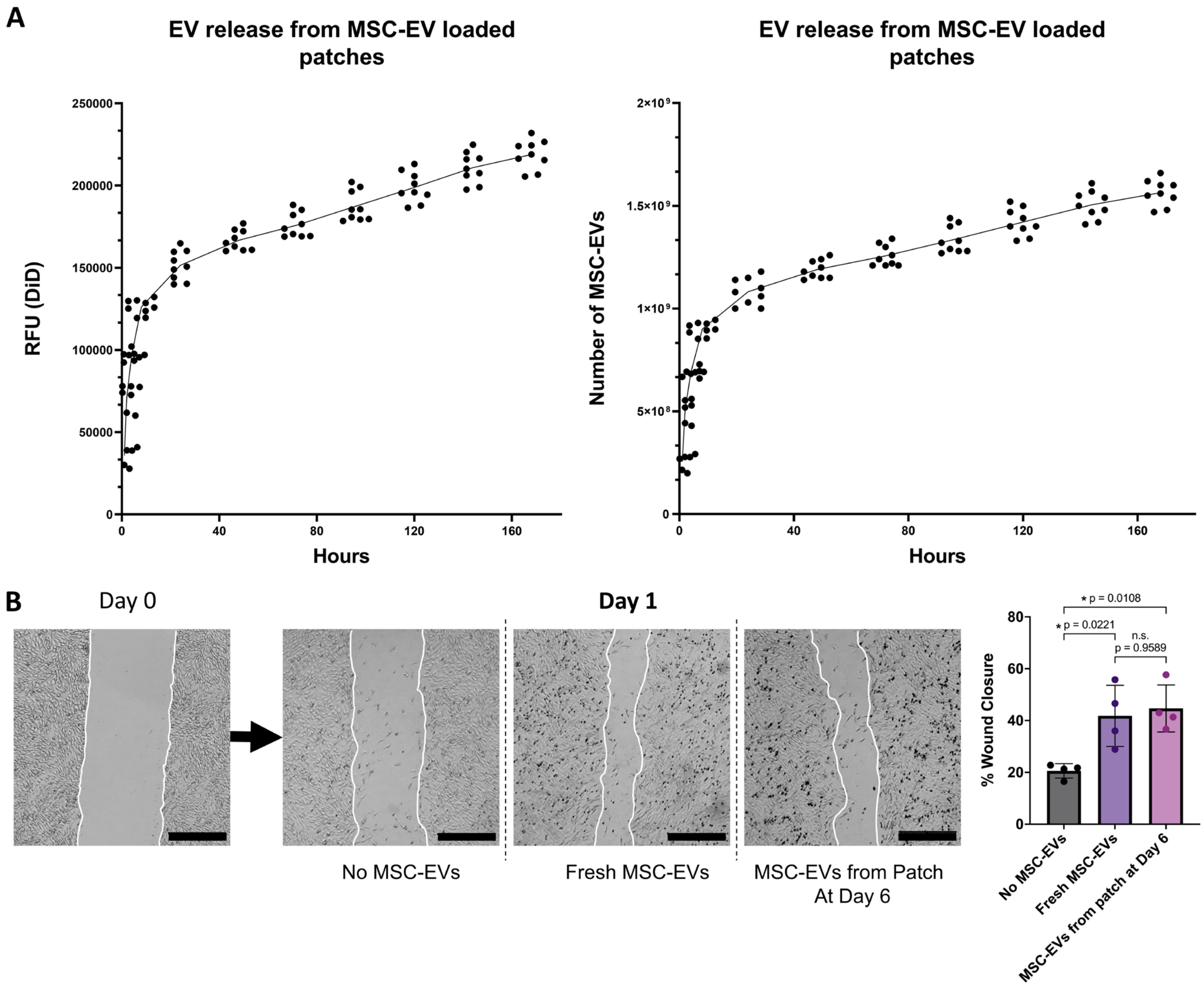

Supplementary Figure 3. A. Release of MSC-EVs from the patches characterized by the fluorescence measurements of the DiD labeling (left) and the corresponding estimation of the number of MSC-EVs being released (right), which demonstrates an initial burst release within the first 12 h, followed by a slow sustained release over a week in culture. **B.** Scratch assays with 3T3 cells (scale bar 500 µm), where the MSC-EVs collected from the patch after a week of incubation demonstrated similar effectiveness for in vitro wound closure when compared to freshly collected MSC-EVs (data analysis was performed using an ImageJ plugin[2]). Statistical significance was analyzed by one-way ANOVA followed by Šídák's multiple comparison test, *p < 0.05. Data in A, B, and E are from n=3 biologically independent samples and are presented as mean ± s.d. Data in A are from n=3 biologically independent samples, and B are from n=4 biologically independent samples and are presented as mean ± s.d.

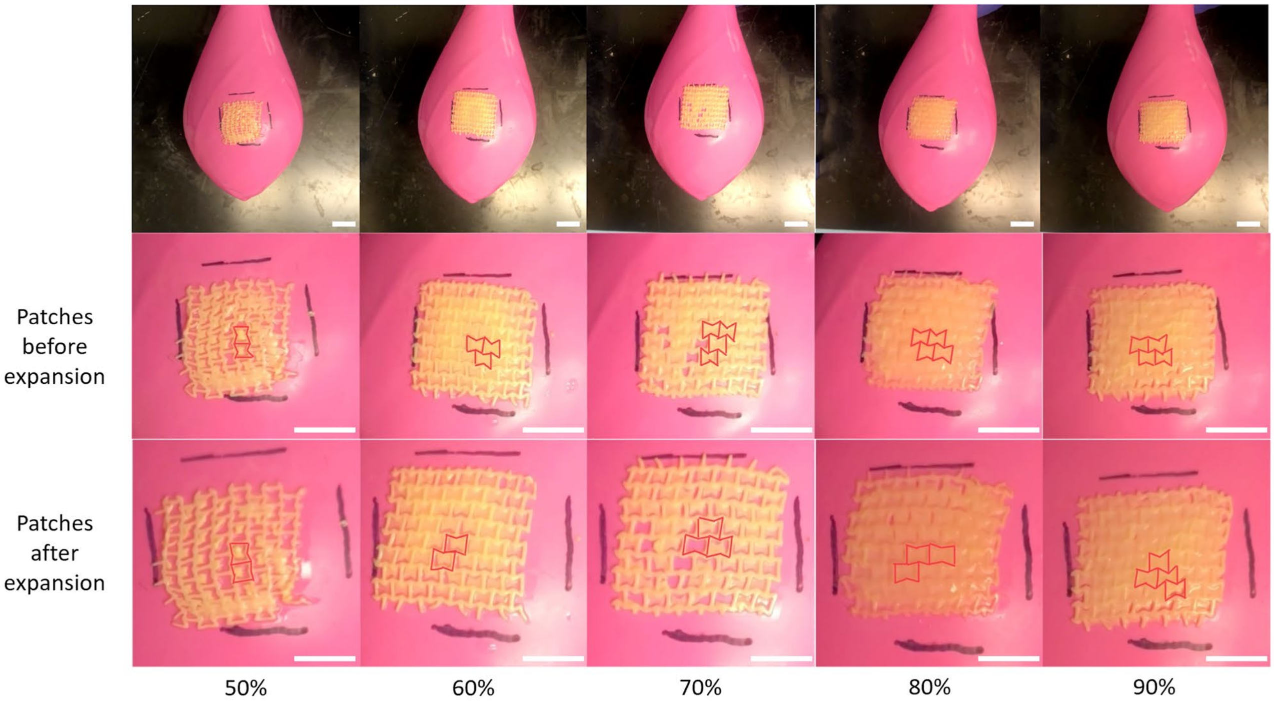

Supplementary Figure 4. Different compositions of the hole-filling material were fabricated under different UV exposure durations and tested for their compliance to the balloon model. The numbers 50-90% at the bottom denote the ink composition as per Table S2. With 15 s crosslinking time, 50% and 60% ink formulation (as per Table S2) cannot crosslink with the original matrix, i.e., the holes cannot be sealed. 70% successfully crosslinked and maintained the auxetic properties, but the hole-filling material was prone to detachment. The ink composition, which had a concentration of the hole-filling material at 80% v/v of that of the lattice material, demonstrated void-filling capability while also exhibiting auxetic characteristics. 90% ink started to cause loss of auxetic property and 100% ink resulted in complete loss of the auxetic property. Scale bar: 10 mm.

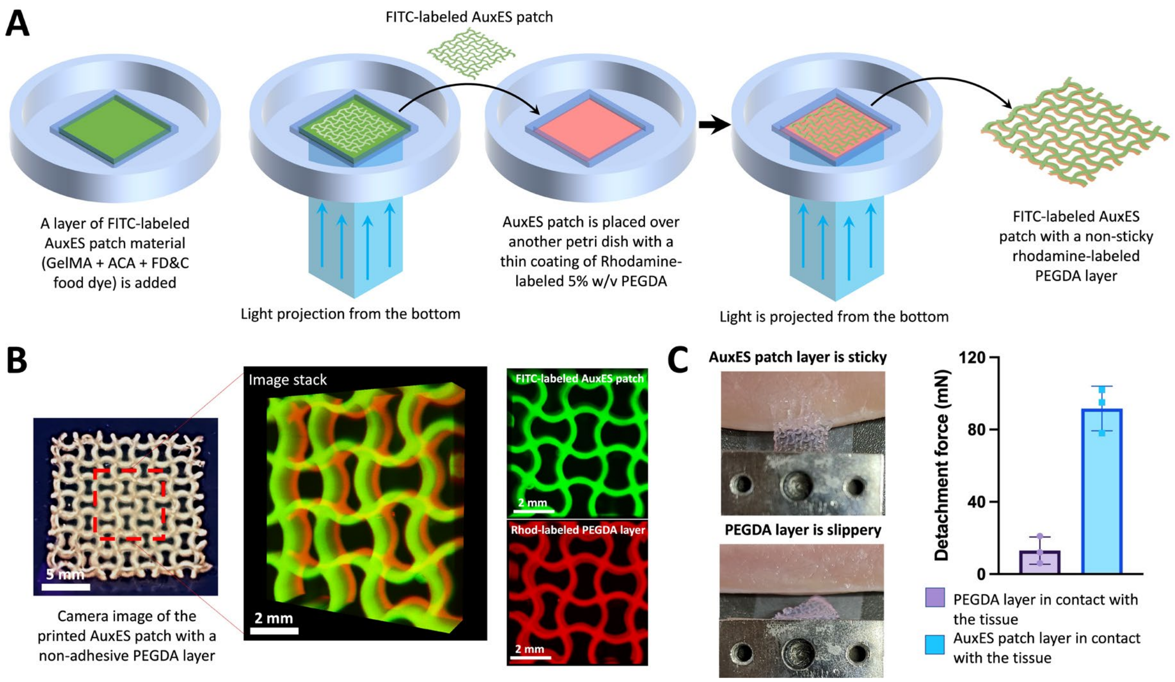

Supplementary Figure 5. A. Scheme of coating a non-adhesive PEGDA layer over the AuxES patches. **B.** fabricated AuxES patches (FITC labeled) with a non-adhesive PEGDA layer (Rhodamine layer). **C.** The patches only demonstrate adhesion via the AuxES patch layer while the PEGDA layer demonstrates negligible adhesion. Data in C are from n=3 biologically independent samples and are presented as mean ± s.d.

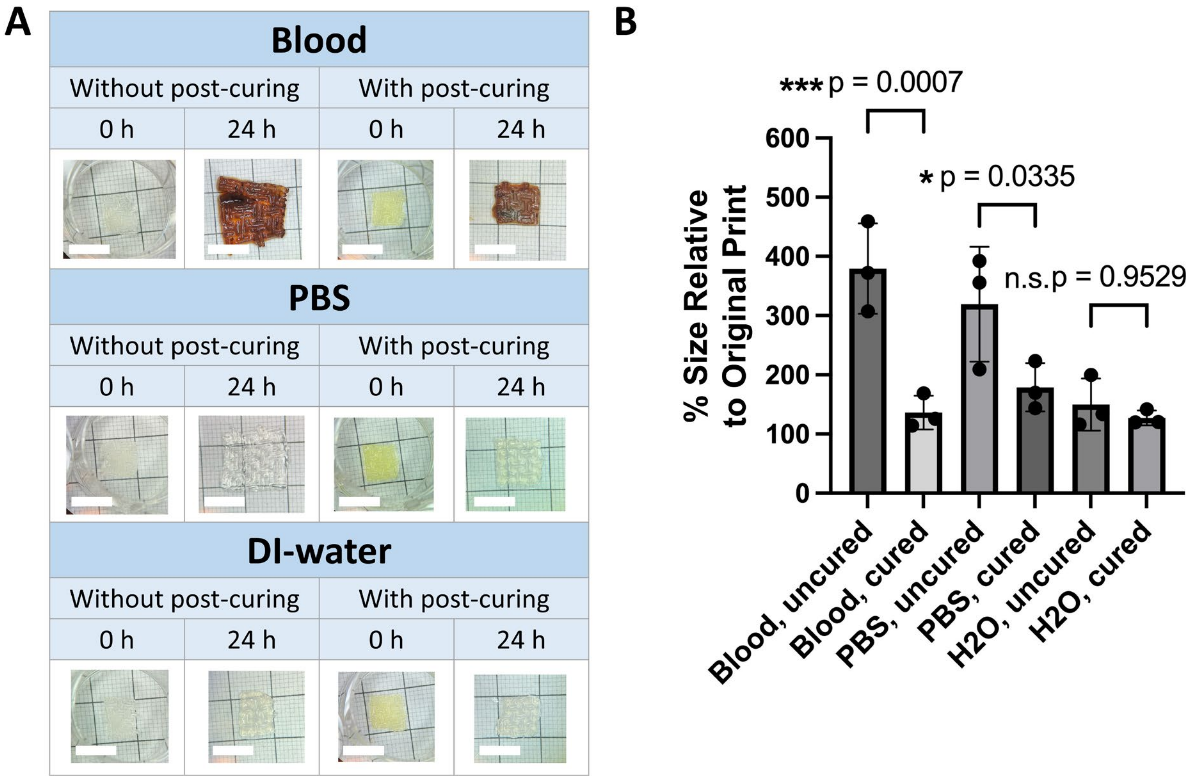

Supplementary Figure 6. Post-curing of patches through additional UV exposure in the presence of photoinitiator after fabrication can mitigate swelling in different media conditions. **A.** Camera images of patches without and with post-curing after incubation in blood, PBS and DI water over 24 h (scale bar: 10 mm). **B.** Corresponding analysis of the size of the patches after swelling relative to the original patch size (100% refers to the patch maintaining its original size upon incubation in the respective media condition). Here, the post-cured patches demonstrated up to 50% and 80% increase in size upon incubation in blood and PBS, respectively, while the uncured patches swelled more than 200% in the respective conditions. A swelling of up to 80% is comparable to the hydrogel systems used for patches. The DI water patches did not demonstrate any swelling, which could be attributed to the absence of competing ions which could disrupt the calcium crosslinks in the patch network. Statistical significance was analyzed by one-way ANOVA followed by Šídák's multiple comparison test, $*p < 0.05$, $**p < 0.01$, and $***p < 0.001$. Data in B are from n=3 biologically independent samples and are presented as mean ± s.d.

Supplementary Table 1. Dimensions of the design elements of the different patch architectures.

Iteration	Geometry	H (mm)	w (mm)	t (mm)	ia (°)	r (mm)	te (mm)
1	Re-entrant honeycomb	3	3	0.75	90	N/A	N/A
2	Re-entrant honeycomb	2.25	2.25	0.75	90	N/A	N/A
3	Re-entrant honeycomb	1.5	1.5	0.75	90	N/A	N/A
4	Re-entrant honeycomb	3	2	0.75	90	N/A	N/A
5	Re-entrant honeycomb	2.25	1.5	0.75	90	N/A	N/A
6	Re-entrant honeycomb	1.5	1	0.75	90	N/A	N/A
7	Re-entrant honeycomb	3	3	1.125	90	N/A	N/A
8	Re-entrant honeycomb	2.25	2.25	1.125	90	N/A	N/A
9	Re-entrant honeycomb	1.5	1.5	1.125	90	N/A	N/A
10	Re-entrant honeycomb	3	2	1.125	90	N/A	N/A
11	Re-entrant honeycomb	2.25	1.5	1.125	90	N/A	N/A
12	Re-entrant honeycomb	1.5	1	1.125	90	N/A	N/A
13	Re-entrant honeycomb	3	3	0.75	112.5	N/A	N/A
14	Re-entrant honeycomb	2.25	2.25	0.75	112.5	N/A	N/A
15	Re-entrant honeycomb	1.5	1.5	0.75	112.5	N/A	N/A
16	Re-entrant honeycomb	3	2	0.75	112.5	N/A	N/A
17	Re-entrant honeycomb	2.25	1.5	0.75	112.5	N/A	N/A
18	Re-entrant honeycomb	1.5	1	0.75	112.5	N/A	N/A
19	Re-entrant honeycomb	3	3	1.125	112.5	N/A	N/A
20	Re-entrant honeycomb	2.25	2.25	1.125	112.5	N/A	N/A
21	Re-entrant honeycomb	1.5	1.5	1.125	112.5	N/A	N/A
22	Re-entrant honeycomb	3	2	1.125	112.5	N/A	N/A
23	Re-entrant honeycomb	2.25	1.5	1.125	112.5	N/A	N/A
24	Re-entrant honeycomb	1.5	1	1.125	112.5	N/A	N/A
1	Lozenge truss	2.25	N/A	0.375	N/A	N/A	0.75

2	Lozenge truss	1.5	N/A	0.375	N/A	N/A	0.75
3	Lozenge truss	3	N/A	0.375	N/A	N/A	0.75
4	Lozenge truss	3	N/A	0.75	N/A	N/A	0.75
5	Lozenge truss	1.5	N/A	0.75	N/A	N/A	0.75
6	Lozenge truss	2.25	N/A	0.75	N/A	N/A	0.75
7	Lozenge truss	3	N/A	0.375	N/A	N/A	1.125
8	Lozenge truss	2.25	N/A	0.75	N/A	N/A	1.125
1	Sinusoidal ligaments	3	3	0.75	N/A	1.875	N/A
2	Sinusoidal ligaments	2.25	2.25	0.75	N/A	1.875	N/A
3	Sinusoidal ligaments	1.5	1.5	0.75	N/A	1.875	N/A
4	Sinusoidal ligaments	3	2	0.75	N/A	1.875	N/A
5	Sinusoidal ligaments	2.25	1.5	0.75	N/A	1.875	N/A
6	Sinusoidal ligaments	1.5	1	0.75	N/A	1.875	N/A
7	Sinusoidal ligaments	3	3	1.125	N/A	1.875	N/A
8	Sinusoidal ligaments	2.25	2.25	1.125	N/A	1.875	N/A
9	Sinusoidal ligaments	3	2	1.125	N/A	1.875	N/A
10	Sinusoidal ligaments	2.25	1.5	1.125	N/A	1.875	N/A
11	Sinusoidal ligaments	1.5	1	1.125	N/A	1.875	N/A
12	Sinusoidal ligaments	3	3	0.75	N/A	2.25	N/A
13	Sinusoidal ligaments	2.25	2.25	0.75	N/A	2.25	N/A
14	Sinusoidal ligaments	1.5	1.5	0.75	N/A	2.25	N/A
15	Sinusoidal ligaments	3	2	0.75	N/A	2.25	N/A
16	Sinusoidal ligaments	2.25	1.5	0.75	N/A	2.25	N/A
17	Sinusoidal ligaments	3	3	1.125	N/A	2.25	N/A
18	Sinusoidal ligaments	2.25	2.25	1.125	N/A	2.25	N/A
19	Sinusoidal ligaments	3	2	1.125	N/A	2.25	N/A

20	Sinusoidal ligaments	2.25	1.5	1.125	N/A	2.25	N/A
21	Sinusoidal ligaments	1.5	1	1.125	N/A	2.25	N/A
1	Honeycomb	3	N/A	0.75	N/A	N/A	N/A
2	Honeycomb	2.25	N/A	0.75	N/A	N/A	N/A
3	Honeycomb	3	N/A	1.125	N/A	N/A	N/A
4	Honeycomb	2.25	N/A	1.125	N/A	N/A	N/A
1	Inclined Truss	3	3	0.75	N/A	N/A	N/A
2	Inclined Truss	2.25	2.25	0.75	N/A	N/A	N/A
3	Inclined Truss	1.5	1.5	0.75	N/A	N/A	N/A
4	Inclined Truss	3	3	1.125	N/A	N/A	N/A
5	Inclined Truss	2.25	2.25	1.125	N/A	N/A	N/A
6	Inclined Truss	1.5	1.5	1.125	N/A	N/A	N/A

Supplementary Table 2. Different compositions of the void-filling material (LAP concentration was the same at 0.03% w/v). In this formulation, the 80% v/v GelMA – 20% v/v ACA mixture (termed as the original ink composition) used for the patch lattices was further diluted to 50, 60, 70, 80, and 90% of its original concentration by mixing with PBS and application to the patch voids followed by UV exposure.

**Composition**	**Amount of Original ink formulation (% v/v) in 1x PBS**	**GelMA (µL)**	**ACA (µL)**	**1x PBS (µL)**
1	100%	500	100	0
2	90%	450	90	60
3	80%	400	80	120
4	70%	350	70	180
5	60%	300	60	240
6	50%	250	50	300

Supplementary Table 3. Statistical data of Nanostring analysis. Statistical analysis of the fold-change values of the different genes was conducted using GraphPad Prism v 9.3.1. Means of the no-patch fold-change were compared to the means of the patch containing CNPs and MSC-EVs using multiple unpaired two-tailed t-tests (one per row). Variance assumptions assumed individual variances for each row, and multiple comparisons were tabulated using False Discovery Rate (FDR), and the two-stage step up method (Benjamini, Krieger, and Yekutieli) with desired Q of 1.00%.

Gene	Discovery	P value	Mean of No Patch	Mean of EVs + Curcumin	Difference	SE of difference	t ratio	df	q value
C2	No	0.310737	1.000	2.129	-1.129	0.9741	1.160	4.000	0.459692
C8G	No	0.888779	1.000	1.079	-0.07923	0.5318	0.1490	4.000	0.897667
C9	No	0.474118	1.000	2.043	-1.043	1.321	0.7892	4.000	0.498207
CAMP	No	0.054254	1.000	0.5100	0.4900	0.1817	2.697	4.000	0.248196
CCR2	No	0.043413	1.000	2.518	-1.518	0.5206	2.916	4.000	0.225810
CCR3	No	0.322673	1.000	2.999	-1.999	1.774	1.127	4.000	0.459692
CCR4	No	0.300292	1.000	2.283	-1.283	1.079	1.189	4.000	0.459692
GPR29	No	0.084662	1.000	2.814	-1.814	0.7952	2.281	4.000	0.307221
ACKR4	No	0.246783	1.000	3.111	-2.111	1.557	1.355	4.000	0.459692
CCRL2	No	0.667334	1.000	1.365	-0.3647	0.7873	0.4632	4.000	0.680615
Cd1d1	No	0.059582	1.000	2.878	-1.878	0.7202	2.607	4.000	0.258261
CD2	No	0.079901	1.000	2.110	-1.110	0.4757	2.334	4.000	0.307221
CD3E	No	0.352579	1.000	2.584	-1.584	1.507	1.051	4.000	0.459692
CD7	No	0.170373	1.000	3.056	-2.056	1.232	1.669	4.000	0.411740
CFB	No	0.406533	1.000	1.688	-0.6875	0.7419	0.9267	4.000	0.459692
CFD	No	0.436346	1.000	2.265	-1.265	1.464	0.8639	4.000	0.472844
CFH°	No	0.027294	1.000	2.427	-1.427	0.4198	3.399	4.000	0.177465
CFI	No	0.354039	1.000	2.775	-1.775	1.695	1.047	4.000	0.459692
CIITA°	No	0.038305	1.000	2.220	-1.220	0.4008	3.043	4.000	0.217268
CLU	No	0.050639	1.000	2.358	-1.358	0.4913	2.764	4.000	0.248196

Gene	Discovery	P value	Mean of No Patch	Mean of EVs + Curcumin	Difference	SE of difference	t ratio	df	q value
CR2	No	0.262282	1.000	2.359	-1.359	1.043	1.304	4.000	0.459692
CSF2	No	0.257003	1.000	2.713	-1.713	1.297	1.321	4.000	0.459692
CTLA4	No	0.381543	1.000	2.653	-1.653	1.683	0.9824	4.000	0.459692
Cxcl15	No	0.384275	1.000	2.743	-1.743	1.786	0.9761	4.000	0.459692
CXCR1	No	0.355534	1.000	2.514	-1.514	1.451	1.044	4.000	0.459692
DEFB1	No	0.078204	1.000	2.018	-1.018	0.4325	2.354	4.000	0.307221
GATA3*	No	0.022575	1.000	2.449	-1.449	0.4016	3.609	4.000	0.174112
GZMB	No	0.260967	1.000	2.520	-1.520	1.162	1.308	4.000	0.459692
ICOS	No	0.412258	1.000	2.118	-1.118	1.222	0.9144	4.000	0.461153
IFNB1	No	0.284856	1.000	3.958	-2.958	2.398	1.234	4.000	0.459692
IL17B	No	0.159041	1.000	3.300	-2.300	1.331	1.728	4.000	0.393930
IL17F	No	0.293902	1.000	2.720	-1.720	1.425	1.207	4.000	0.459692
IL18	No	0.139785	1.000	2.785	-1.785	0.9707	1.839	4.000	0.368184
IL2	No	0.210897	1.000	3.110	-2.110	1.418	1.488	4.000	0.459692
IL21	No	0.405717	1.000	2.496	-1.496	1.611	0.9285	4.000	0.459692
IL23R	No	0.349195	1.000	2.003	-1.003	0.9467	1.059	4.000	0.459692
IL2RB°	No	0.005319	1.000	2.251	-1.251	0.2274	5.503	4.000	0.129937
IL5	No	0.174148	1.000	2.874	-1.874	1.136	1.651	4.000	0.411740
ILF3	No	0.158883	1.000	2.788	-1.788	1.034	1.729	4.000	0.393930
KIR3DL1	No	0.362668	1.000	0.4170	0.5830	0.5680	1.027	4.000	0.459692
KIR3DL2	No	0.447744	1.000	2.144	-1.144	1.360	0.8409	4.000	0.475294
KLRD1	No	0.097358	1.000	2.505	-1.505	0.6982	2.156	4.000	0.316505
Klra4	No	0.207935	1.000	3.344	-2.344	1.562	1.500	4.000	0.459692
Klra6	No	0.290227	1.000	3.956	-2.956	2.428	1.218	4.000	0.459692

Gene	Discovery	P value	Mean of No Patch	Mean of EVs + Curcumin	Difference	SE of difference	t ratio	df	q value
Klra7	No	0.340173	1.000	2.774	-1.774	1.640	1.082	4.000	0.459692
Klra8**	No	0.000105	1.000	1.936	-0.9358	0.06102	15.34	4.000	0.010969
Klrd1	No	0.132000	1.000	2.532	-1.532	0.8114	1.888	4.000	0.368184
LAIR1	No	0.648755	1.000	1.299	-0.2995	0.6092	0.4916	4.000	0.668217
LILRA6	No	0.243468	1.000	2.216	-1.216	0.8893	1.367	4.000	0.459692
MASP2	No	0.445766	1.000	2.132	-1.132	1.340	0.8449	4.000	0.475294
MBL2	No	0.360758	1.000	2.500	-1.500	1.454	1.031	4.000	0.459692
MUC1	No	0.097352	1.000	0.4594	0.5406	0.2508	2.156	4.000	0.316505
MX1	No	0.398248	1.000	3.171	-2.171	2.297	0.9448	4.000	0.459692
NFATC2*	No	0.012490	1.000	2.159	-1.159	0.2686	4.316	4.000	0.129937
NFKBIZ	No	0.138045	1.000	0.4918	0.5082	0.2748	1.850	4.000	0.368184
NOTCH1*	No	0.012177	1.000	2.097	-1.097	0.2524	4.348	4.000	0.129937
NOX3	No	0.431643	1.000	2.101	-1.101	1.261	0.8736	4.000	0.472672
Pdcd1lg2	No	0.405976	1.000	2.347	-1.347	1.452	0.9279	4.000	0.459692
PDCD1LG2	No	0.114492	1.000	3.148	-2.148	1.067	2.012	4.000	0.350313
SELPLG*	No	0.010164	1.000	2.588	-1.588	0.3466	4.583	4.000	0.129937
STAT2	No	0.398504	1.000	1.409	-0.4086	0.4327	0.9443	4.000	0.459692
STAT6*	No	0.010581	1.000	2.006	-1.006	0.2220	4.529	4.000	0.129937
TCF7	No	0.110819	1.000	2.717	-1.717	0.8411	2.041	4.000	0.349349
TNFRSF11A	No	0.335217	1.000	2.823	-1.823	1.665	1.095	4.000	0.459692
TNFRSF17	No	0.395986	1.000	2.470	-1.470	1.547	0.9498	4.000	0.459692
TNFSF11*	No	0.039682	1.000	2.275	-1.275	0.4239	3.007	4.000	0.217268
TNFSF15	No	0.361226	1.000	2.343	-1.343	1.304	1.030	4.000	0.459692
TNFSF18	No	0.381904	1.000	2.260	-1.260	1.283	0.9815	4.000	0.459692

Gene	Discovery	P value	Mean of No Patch	Mean of EVs + Curcumin	Difference	SE of difference	t ratio	df	q value
XCR1	No	0.141568	1.000	2.260	-1.260	0.6892	1.828	4.000	0.368184
CASP2*	No	0.024736	1.000	2.658	-1.658	0.4729	3.507	4.000	0.174112
CCL19	No	0.141456	1.000	2.111	-1.111	0.6073	1.829	4.000	0.368184
CD19	No	0.294251	1.000	3.258	-2.258	1.872	1.206	4.000	0.459692
CD24	No	0.003966	1.000	2.780	-1.780	0.2984	5.965	4.000	0.129937
CD55	No	0.085880	1.000	1.869	-0.8688	0.3830	2.268	4.000	0.307221
CD79A	No	0.257877	1.000	2.961	-1.961	1.488	1.318	4.000	0.459692
CX3CL1*	No	0.024928	1.000	2.087	-1.087	0.3108	3.499	4.000	0.174112
CXCL13	No	0.123192	1.000	3.396	-2.396	1.230	1.948	4.000	0.366162
CXCR6	No	0.238941	1.000	2.132	-1.132	0.8184	1.383	4.000	0.459692
GFI1	No	0.500916	1.000	1.769	-0.7690	1.041	0.7390	4.000	0.521103
IFNG	No	0.309422	1.000	2.088	-1.088	0.9354	1.163	4.000	0.459692
IL10	No	0.372960	1.000	2.195	-1.195	1.193	1.002	4.000	0.459692
IL11	No	0.054874	1.000	2.307	-1.307	0.4866	2.686	4.000	0.248196
MAP4K1	No	0.246877	1.000	2.333	-1.333	0.9834	1.355	4.000	0.459692
MAPK11	No	0.270403	1.000	1.903	-0.9030	0.7066	1.278	4.000	0.459692
MS4A1	No	0.431117	1.000	2.055	-1.055	1.206	0.8747	4.000	0.472672
Plau*	No	0.025105	1.000	2.035	-1.035	0.2966	3.491	4.000	0.174112
Prdm1**	No	0.008173	1.000	2.798	-1.798	0.3686	4.878	4.000	0.129937
RAE1	No	0.088596	1.000	2.139	-1.139	0.5086	2.240	4.000	0.307221
TYK2	No	0.258352	1.000	3.266	-2.266	1.721	1.317	4.000	0.459692
ADAL	No	0.253394	1.000	3.751	-2.751	2.063	1.333	4.000	0.459692
AICDA	No	0.310537	1.000	2.151	-1.151	0.9918	1.160	4.000	0.459692
DPP4	No	0.189521	1.000	1.375	-0.3751	0.2376	1.579	4.000	0.438131

Gene	Discovery	P value	Mean of No Patch	Mean of EVs + Curcumin	Difference	SE of difference	t ratio	df	q value
FOLR4	No	0.274663	1.000	2.239	-1.239	0.9797	1.265	4.000	0.459692
ICAM1	No	0.404114	1.000	2.404	-1.404	1.506	0.9320	4.000	0.459692
IL19	No	0.287643	1.000	2.799	-1.799	1.468	1.225	4.000	0.459692
IL25	No	0.357345	1.000	2.257	-1.257	1.210	1.039	4.000	0.459692
IL33*	No	0.012223	1.000	2.068	-1.068	0.2459	4.343	4.000	0.129937
IL4	No	0.401930	1.000	2.333	-1.333	1.423	0.9367	4.000	0.459692
ITGA2B	No	0.299923	1.000	3.294	-2.294	1.928	1.190	4.000	0.459692
ITGA6	No	0.015643	1.000	2.073	-1.073	0.2657	4.037	4.000	0.147938
LTBR**	No	0.006280	1.000	2.344	-1.344	0.2559	5.254	4.000	0.129937
Pdgfb	No	0.075183	1.000	2.073	-1.073	0.4488	2.390	4.000	0.307221
ZBTB7B	No	0.036957	1.000	2.417	-1.417	0.4603	3.079	4.000	0.217268

Supplementary References:

1. Chansoria, P., Etter, E. L. & Nguyen, J. Regenerating dynamic organs using biomimetic patches. *Trends Biotechnol.* (2021) doi:10.1016/J.TIBTECH.2021.07.001.
2. Suarez-Arnedo, A. *et al.* An image J plugin for the high throughput image analysis of in vitro scratch wound healing assays. *PLoS One* **15**, (2020).
