# Supplementary material for: Instantly adhesive and ultra-elastic patches for dynamic organ and wound repair
Source: Nat Commun. 2024 Jun 3;15:4720. doi: 10.1038/s41467-024-48980-0 (PMC11148085; doi:10.1038/s41467-024-48980-0)
Supplement: Supplementary file 3 — Description of Additional Supplementary Files [file 41467_2024_48980_MOESM3_ESM.pdf]

Description of Additional Supplementary Files

Title: Supplementary Movie 1
Description: Adhesion of the AuxES patches to a wet organ.

Title: Supplementary Movie 2
Description: Patches treated with calcium chloride solution display enhanced adhesion to wet tissue such as the liver.

Title: Supplementary Movie 3
Description: The AuxES patch formulation possesses ultra-elasticity.

Title: Supplementary Movie 4
Description: AuxES patches conform to balloon expansion and contraction.

Title: Supplementary Movie 5
Description: The auxetic lozenge truss patch (right) demonstrated a greater increase in overall surface area during physiological ventilation (PV) and hyperventilation (HV) states compared with the non-auxetic honeycomb patch (left). This ex vivo experiment was performed on porcine lungs.

Title: Supplementary Movie 6
Description: AuxES patches can comply with the rapid motion of the heart and demonstrate rhythmic stretching and contraction in synchrony with the diastolic and systolic cycles of the heart. This in vivo experiment was performed on a beating rodent heart.

Title: Supplementary Movie 7
Description: Conformation of the AuxES patches to the movement of the foot during dorsiflexion and plantarflexion in an anatomical foot model. Re-en itr1 is most suitable for the sole while Re-en itr4 is most suitable for the dorsum.

Title: Supplementary Movie 8
Description: Void-filled AuxES patches retain their auxetic nature and conform with the auxetic mechanics of the balloon's inflation and deflation.

Title: Supplementary Movie 9
Description: Instantaneous and strong adhesiveness of the void-filled AuxES patches inhibits pulmonary air leakage in a rat model.

Title: Supplementary Movie 10
Description: AuxES patches demonstrate bioadhesive properties after exposure to blood and PBS.
