# Supplementary material for: Instantly adhesive and ultra-elastic patches for dynamic organ and wound repair
Source: Nat Commun. 2024 Jun 3;15:4720. doi: 10.1038/s41467-024-48980-0 (PMC11148085; doi:10.1038/s41467-024-48980-0)
Supplement: Supplementary file 14 — Reporting Summary [file 41467_2024_48980_MOESM14_ESM.pdf]

nature portfolio

Corresponding author(s): Dr. Juliane Nguyen

Last updated by author(s): Apr 19, 2024

Reporting Summary

Nature Portfolio wishes to improve the reproducibility of the work that we publish. This form provides structure for consistency and transparency in reporting. For further information on Nature Portfolio policies, see our Editorial Policies and the Editorial Policy Checklist.

Statistics

For all statistical analyses, confirm that the following items are present in the figure legend, table legend, main text, or Methods section.

n/a	Confirmed	
☐	☒	The exact sample size (*n*) for each experimental group/condition, given as a discrete number and unit of measurement
☐	☒	A statement on whether measurements were taken from distinct samples or whether the same sample was measured repeatedly
☐	☒	The statistical test(s) used AND whether they are one- or two-sided<br>*Only common tests should be described solely by name; describe more complex techniques in the Methods section.*
☒	☐	A description of all covariates tested
☐	☒	A description of any assumptions or corrections, such as tests of normality and adjustment for multiple comparisons
☐	☒	A full description of the statistical parameters including central tendency (e.g. means) or other basic estimates (e.g. regression coefficient) AND variation (e.g. standard deviation) or associated estimates of uncertainty (e.g. confidence intervals)
☐	☒	For null hypothesis testing, the test statistic (e.g. *F*, *t*, *r*) with confidence intervals, effect sizes, degrees of freedom and *P* value noted<br>*Give P values as exact values whenever suitable.*
☒	☐	For Bayesian analysis, information on the choice of priors and Markov chain Monte Carlo settings
☒	☐	For hierarchical and complex designs, identification of the appropriate level for tests and full reporting of outcomes
☒	☐	Estimates of effect sizes (e.g. Cohen's *d*, Pearson's *r*), indicating how they were calculated

Our web collection on statistics for biologists contains articles on many of the points above.

Software and code

Policy information about availability of computer code

Data collection	COMSOL v5.3a, FIJI ImageJ v2.14.0/1.54f, Solidworks 2021
Data analysis	GraphPad PRISM 10, Microsoft Excel (Version 16.79.1), nSolver v4.0, JMP v17.2.0

For manuscripts utilizing custom algorithms or software that are central to the research but not yet described in published literature, software must be made available to editors and reviewers. We strongly encourage code deposition in a community repository (e.g. GitHub). See the Nature Portfolio guidelines for submitting code & software for further information.

Data

Policy information about availability of data

All manuscripts must include a data availability statement. This statement should provide the following information, where applicable:

- Accession codes, unique identifiers, or web links for publicly available datasets
- A description of any restrictions on data availability
- For clinical datasets or third party data, please ensure that the statement adheres to our policy

Data from experiments conducted in this study are provided in the Article and Supplementary Information. Pathway enrichment analysis was performed using Panther and Reactome databases from pantherdb.org. The data underlying Fig. 1B, C; Fig. 2B; Fig. 3A; Fig. 4C-F; Fig. 5C,D; Fig. 6A, and Fig 7B,E as well as Supplementary Figs. 1-3, 5, 6 are in the associated Source Data file. All data are available from the authors upon request.

Research involving human participants, their data, or biological material

Policy information about studies with human participants or human data. See also policy information about sex, gender (identity/presentation), and sexual orientation and race, ethnicity and racism.

Reporting on sex and gender	n/a
Reporting on race, ethnicity, or other socially relevant groupings	n/a
Population characteristics	n/a
Recruitment	n/a
Ethics oversight	n/a

Note that full information on the approval of the study protocol must also be provided in the manuscript.

Field-specific reporting

Please select the one below that is the best fit for your research. If you are not sure, read the appropriate sections before making your selection.

[x] Life sciences   [ ] Behavioural & social sciences   [ ] Ecological, evolutionary & environmental sciences

For a reference copy of the document with all sections, see nature.com/documents/nr-reporting-summary-flat.pdf

Life sciences study design

All studies must disclose on these points even when the disclosure is negative.

Sample size	For all the experiments, a minimum sample size of 3 biologically independent samples was used to assess statistical significance. For the wound healing experiment, an initial pilot study was performed with n=4 (one wound per mouse). Based on the measured outcome, it was determined that to have 90% power while demonstrating statistically significant difference, n=8 per group was required.
Data exclusions	No replicates were excluded from the study.
Replication	For the patch optimization, cell assays, ex vivo experiments, and pulmonary air leakage model, each experiment was repeated at least once. The wound healing experiment was reliably reproduced thrice and performed independently.
Randomization	All experiments had random allocation of samples/mices/ rats.
Blinding	Material synthesis and patch fabrication was not blinded so that investigators could follow different protocols meticulously. Due the nature of our patch system blinding was not possible in any experiments because treatment groups were easily identified due to the presence and appearance of the treatment (AuxES patch). Therefore blinding was not possible. Histological analysis was blinded.

Reporting for specific materials, systems and methods

We require information from authors about some types of materials, experimental systems and methods used in many studies. Here, indicate whether each material, system or method listed is relevant to your study. If you are not sure if a list item applies to your research, read the appropriate section before selecting a response.

Materials & experimental systems

n/a	Involved in the study	
[x]	[ ]	Antibodies
[ ]	[x]	Eukaryotic cell lines
[x]	[ ]	Palaeontology and archaeology
[ ]	[x]	Animals and other organisms
[x]	[ ]	Clinical data
[x]	[ ]	Dual use research of concern
[x]	[ ]	Plants

Methods

n/a	Involved in the study	
[x]	[ ]	ChIP-seq
[x]	[ ]	Flow cytometry
[x]	[ ]	MRI-based neuroimaging

Eukaryotic cell lines

Policy information about cell lines and Sex and Gender in Research

Cell line source(s)	Human bone-marrow Mensenchymal Stem Cells (ATCC PCS-500-012) were purchased from American Type Culture Collection (ATCC, Manassas, VA). NIH/3T3 (ATCC CRL-1658) mouse fibroblast cells were purchased from ATCC via Tissue Culture Facility at University of North Carolina, Chapel Hill.
Authentication	All cell lines are commercially available and were validated by the manufacturer. We did not independently authenticate them.
Mycoplasma contamination	Cell lines were directly purchased from the manufacturer and based on the certificate of analysis mycoplasma was not detected.
Commonly misidentified lines (See ICLAC register)	Commonly misidentified cell lines were not used.

Animals and other research organisms

Policy information about studies involving animals; ARRIVE guidelines recommended for reporting animal research, and Sex and Gender in Research

Laboratory animals	Female, 6-8 week old C57BL/6J were purchased from Jackson Laboratory. Male, 12 weeks old Sprague Dawley (SD) rats were purchased from Charles River Laboratories. The mice and rats were housed at ~21°C and ~50% relative humidity with 12 h light–dark cycles and had access to food and water.
Wild animals	Wild animals were not used.
Reporting on sex	No report on sex differences was found.
Field-collected samples	Field-collected samples were not involved.
Ethics oversight	All in vivo experiments were conducted under the guidance and in compliance with regulations of Institutional Animal Care and Use Committee (IACUC) of University of North Carolina, Chapel Hill under protocol 20-045.0, 23-029.0 and 21-268.0.

Note that full information on the approval of the study protocol must also be provided in the manuscript.

Plants

Seed stocks	n/a
Novel plant genotypes	n/a
Authentication	n/a
